# Untangling glycaemia and mortality in critical care

**DOI:** 10.1186/s13054-017-1725-y

**Published:** 2017-06-24

**Authors:** Vincent Uyttendaele, Jennifer L. Dickson, Geoffrey M. Shaw, Thomas Desaive, J. Geoffrey Chase

**Affiliations:** 1 Department of Mechanical Engineering, University of Canterbury, Private Bag 4800, Christchurch, New Zealand; 2 Department of Intensive Care, Christchurch Hospital, Private Bag 4710, Christchurch, New Zealand; 3 GIGA – In Silico Medicine, University of Liège, Allée du 6 Août 19, bâtiment B5a, 4000 Liège, Belgium

**Keywords:** Critical care, Hyperglycaemia, Glycaemic control, Blood glucose, Variability, Insulin sensitivity, Insulin

## Abstract

**Background:**

Hyperglycaemia is associated with adverse outcomes in the intensive care unit, and initial studies suggested outcome benefits of glycaemic control (GC). However, subsequent studies often failed to replicate these results, and they were often unable to achieve consistent, safe control, raising questions about the benefit or harm of GC as well as the nature of the association of glycaemia with mortality and clinical outcomes. In this study, we evaluated if non-survivors are harder to control than survivors and determined if glycaemic outcome is a function of patient condition and eventual outcome or of the glycaemic control provided.

**Methods:**

Clinically validated, model-based, hour-to-hour insulin sensitivity (SI) and its hour-to-hour variability (%ΔSI) were identified over the first 72 h of therapy in 145 patients (119 survivors, 26 non-survivors). In hypothesis testing, we compared distributions of SI and %ΔSI in 6-hourly blocks for survivors and non-survivors. In equivalence testing, we assessed if differences in these distributions, based on blood glucose measurement error, were clinically significant.

**Results:**

SI level was never equivalent between survivors and non-survivors (95% CI of percentage difference in medians outside ±12%). Non-survivors had higher SI, ranging from 9% to 47% higher overall in 6-h blocks, and this difference became statistically significant as glycaemic control progressed. %ΔSI was equivalent between survivors and non-survivors for all 6-hourly blocks (95% CI of difference in medians within ±12%) and decreased in general over time as glycaemic control progressed.

**Conclusions:**

Whereas non-survivors had higher SI levels, variability was equivalent to that of survivors over the first 72 h. These results indicate survivors and non-survivors are equally controllable, given an effective glycaemic control protocol, suggesting that glycaemia level and variability, and thus the association between glycaemia and outcome, are essentially determined by the control provided rather than by underlying patient or metabolic condition.

**Electronic supplementary material:**

The online version of this article (doi:10.1186/s13054-017-1725-y) contains supplementary material, which is available to authorized users.

## Background

### Rationale

Glycaemic control (GC) in the intensive care unit (ICU) is a controversial subject [1–7]. Whereas some studies showed improved mortality with GC within a tight or intermediate range [8–12], several others studies and larger analyses did not reproduce these results [13–23]. Increased hypoglycaemia induced by the GC protocol, patient variability and/or protocol compliance further confounds results.

The strong associations of blood glucose (BG) level and/or variability with mortality [24–31] have been used to make a case for GC. The association of moderate or severe hypoglycaemia with increased mortality [29, 32–34] similarly indicates that improved control must be achieved safely, despite high inter- and intra- patient variability [28, 31, 35–39]. The association of high times in intermediate bands with reduced mortality [40–45] would indicate that this control quality must be consistent over time and for most (or all) patients, which was achieved in only a few studies considering outcome [8–10, 12]. This overall case states that outcomes are driven largely by the quality and consistency of GC.

However, association is not causality. Another, equally valid interpretation of these associations is that non-survivors are harder to control, and thus they have the higher glycaemic levels and variability associated with mortality. Similarly, it may be that patients who die are more variable and are thus more likely, under insulin control, to experience moderate or severe hypoglycaemia as a result of their underlying metabolic variability. Such patients would also have less time in intermediate bands. The equivalent case states that survivors are less variable and thus easier to control, resulting in the more normal, consistent glycaemia associated with improved outcomes. This overall case suggests glycaemia and outcomes are driven by patient condition, regardless of GC protocol, or even that ineffective GC causes harm [5].

Separating these two interpretations would clarify the debate, research and practice in GC. In the first case, do we need better control, including any new sensors and devices, to achieve safe, effective and consistent GC for all patients in any unit? Or, in the second case, are GC and its outcomes merely a reflection of the underlying patient state and thus perhaps less necessary to control beyond a modest lowering? In summary, are patient glycaemia and outcome (predominantly) a function of the GC achieved, or are they driven by patient condition?

### Aim and research question

The aim of this study was to separate these two interpretations by asking the question, Are patients who die harder (metabolically) to control than patients who live? If they are harder to control, then it could be considered that patient condition drives glycaemia and outcome. If not, then the quality of control could have the greater influence.

This question is addressed through a retrospective analysis of clinical data and metabolic level and variability using a clinically validated metabolic model [46–49]. Lower metabolic level, captured as lower insulin sensitivity (SI), indicates that increased insulin is required to lower BG, which increases hypoglycaemic risk if there is variability. Greater metabolic variability, captured as greater hour-to-hour percentage change in insulin sensitivity (%ΔSI), translates to greater outcome glycaemic variability in response to insulin. Thus, both measures capture the level of difficulty in GC, where a constant level of SI could be readily titrated to an optimal insulin dose, but unpredictable patient variability can result in excessive hyper- and hypo- glycaemia and glycaemic variability.

In short, do non-survivors have lower SI and/or greater %ΔSI, indicative of being harder to control than survivors? A positive answer would indicate that the well-known associations between glycaemia and outcome are driven more predominantly by patient condition. If non-survivors were similarly difficult or easier to control than survivors, it would indicate that the quality of GC achieved predominates in determining glycaemia and outcome.

## Methods

To answer the research question, metabolic state and variability were analysed using model-based SI. Key outcomes included the following:

- Difference and/or equivalence of SI in survivors and non-survivors
- Difference and/or equivalence of %ΔSI in survivors and non-survivors

These outcomes are compared in 6-h blocks across the first 72 h of patient GC in the ICU.

### Patient cohort

**Fig. 1:**
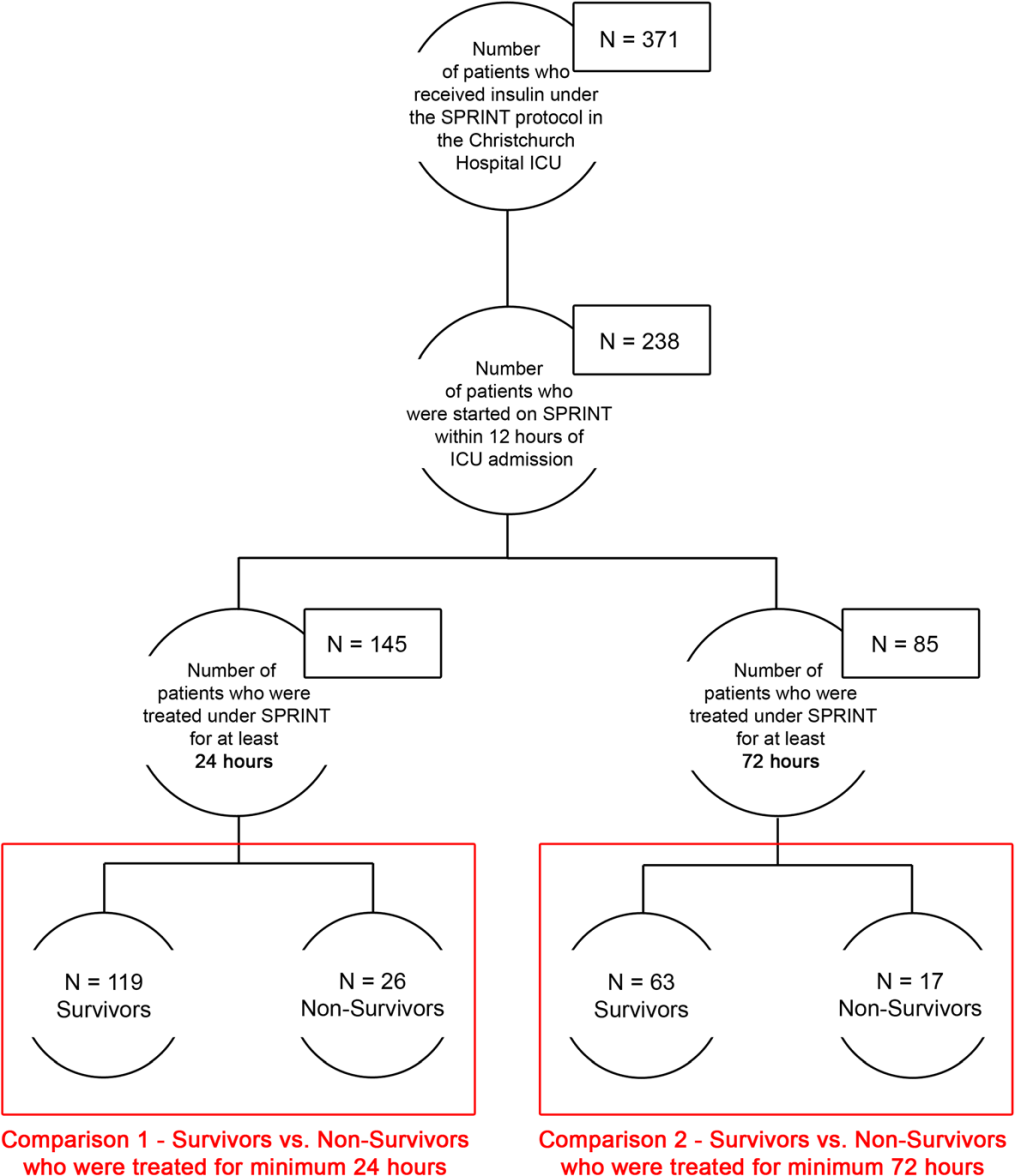
Cohort selection from original 371 patients who treated according to the Specialised Relative Insulin Nutrition Tables (SPRINT) protocol. The first comparison compares survivors and non-survivors from Cohort 1, using as much data as possible and excluding patients with a very short intensive care unit (ICU) stay. The second comparison uses Cohort 2 to assess the impact of competing risk due to patient dropout

Retrospective clinical data from 371 patients on the Specialised Relative Insulin Nutrition Tables (SPRINT) GC protocol in the Christchurch Hospital Department of Intensive Care between August 2005 and April 2007 [10] were analysed. The SPRINT protocol modulated both insulin and nutrition, averaging approximately 16 BG measures per day. Figure 1 shows the inclusion criteria for study analysis. Of 371 patients, 231 patients were started on SPRINT within 12 h of ICU admission, and 145 underwent at least 24 h of insulin therapy. These patients make up Cohort 1, with demographic data listed in Table 1.

**Table 1:** Baseline data of Cohort 1, comprising 145 patients treated according to Specialised Relative Insulin Nutrition Tables protocol

	Cohort 1	Survivors	Non-survivors	*p* Value
Number of subjects	145	119 (82%)	26 (18%)	
Age, years	67 [57–75]	66 [57–74]	73 [59–78]	0.15
Sex, M/F	91/54	75/44	16/10	1.00
APACHE II score	20 [17–26]	19 [16–25]	22 [19–31]	<0.01
First-day SOFA score	6 [4–8]	6 [4–8]	8 [6–8]	0.02
Cardiac	3 [1–4]	3 [1–4]	4 [1–4]	
Pulmonary	3 [2–4]	3 [2–3]	3 [2–4]	
Hepatic	0 [0–0]	0 [0–0]	0 [0–1]	
Renal	0 [0–0]	0 [0–0]	0 [0–0]	
Coagulation	0 [0–1]	0 [0–1]	0 [0–0]	
ICU length of stay, h	113 [65–212]	127 [65–256]	108 [65–154]	0.49
SPRINT duration, h	83 [44–159]	81 [42–168]	101.5 [55–126]	0.93
Diabetes mellitus type 1/type 2, % of total	9/24 (33)	8/21 (29)	1/3 (4)	1.00
Cohort BG, mmol/L	5.7 [4.9–6.7]	5.8 [5.0–6.8]	5.5 [4.8–6.4]	<0.01^a^
Per-patient BG, mmol/L	5.7 [5.2–6.2]	5.8 [5.2–6.2]	5.3 [5.1–5.9]	0.03
Per-patient % BG 4.4–8 mmol/L (% all BG)	82.8 [71.9–89.5] (79.3)	82.1 [72.2–89.3] (79.1)	83.3 [70.4–94.4] (80.0)	0.71
Per-patient % BG <4 mmol/L (% all BG)	1.4 [0.0–5.6] (3.4)	1.4 [0.0–4.2] (3.0)	1.9 [0.0–8.5] (5.0)	0.19
Patients with BG <2.2 mmol/L, *n*	0	0	0	
BG measurements per day	15.8 [14.5–17.7]	15.8 [14.4–18.0]	15.7 [14.8–16.2]	0.80
Per-patient median insulin, U/h	3 [2–3]	3 [2–3]	3 [2–3]	0.34
Per-patient median feed, g/h	3.2 [1.9–4.8]	3.3 [1.9–4.5]	3.1 [2.0–5.3]	0.58

Data are given as median [IQR] unless otherwise indicated. *p* Values were computed using Fisher’s exact text and rank-sum tests where appropriate

*Abbreviations: APACHE* Acute Physiology and Chronic Health Evaluation, *BG* Blood glucose, *ICU* Intensive care unit, *SOFA* Sequential Organ Failure Assessment, *SPRINT* Specialised Relative Insulin Nutrition Tables protocol

^a^Equivalence, as explained in ‘Analyses and statistics’ subsection under ‘Methods’

Glycaemically, survivors and non-survivors had similar times in band. The cohort median BG was statistically different (5.8 vs 5.5 mmol/L, *p* < 0.01), but this difference is within clinical equivalence (explained in ‘Analyses and statistics’ subsection below) and thus considered not clinically significant. Maximum Sequential Organ Failure Assessment scores on Day 1, excluding Glasgow Coma Scale score [50], were higher for non-survivors, as expected, and detailed breakdowns for specific co-morbidities showed similar trends. All other demographics are similar, except for an expected difference in Acute Physiology and Chronic Health Evaluation II score.

To assess any impact of patient dropout, in Cohort 2, we considered only patients who underwent at least 72 h of GC (80 patients). In the first cohort, we assessed as much data as possible, excluding patients with very short ICU stays, whereas in the second cohort, we assessed the impact of competing risk in the analysis of SI and mortality outcome due to patient dropout. Demographic data of Cohort 2 are shown in Table 2 and are similar to those of Cohort 1.

**Table 2:** Baseline data from Cohort 2, comprising 80 patients treated according to Specialised Relative Insulin Nutrition Tables protocol

	Cohort 2	Survivors	Non-survivors	*p* Value
Number of subjects	80	63	17	
Age, years	66 [54–75]	65 [49–74]	73 [57–76]	0.50
Sex, M/F	51/29	41/22	10/7	0.78
APACHE II score	21 [17–27]	21 [16–27]	21 [17–28]	0.60
First-day SOFA score	7 [4–8]	6 [4–8]	8 [6–8]	0.11
Cardiac	3 [1–4]	3 [1–4]	4 [2–4]	
Pulmonary	3 [2–4]	3 [2–4]	4 [2–4]	
Hepatic	0 [0–0]	0 [0–0]	0 [0–1]	
Renal	0 [0–0]	0 [0–0]	0 [0–0]	
Coagulation	0 [0–1]	0 [0–1]	0 [0–1]	
ICU length of stay, h	180 [136–371]	214 [142–405]	142 [108–159]	<0.01
SPRINT duration, h	155 [109–301]	161 [126–332]	110 [102–151]	0.01
Diabetes mellitus type 1/type 2, % of total	5/10 (15)	4/8 (12)	1/2 (3)	1.00
Cohort BG, mmol/L	5.7 [5.0–6.7]	5.8 [5.1–6.8]	5.6 [4.9–6.5]	<0.01^a^
Per-patient BG, mmol/L	5.8 [5.3–6.2]	5.9 [5.4–6.2]	5.4 [5.2–6.0]	0.11
Per-patient % BG, 4.4–8 mmol/L, % of all BG	84.7 [73.6–91.7] (81.3)	84.7 [74.0–91.7] (81.5)	83.3 [71.5–94.8] (80.7)	0.98
Per-patient % BG <4 mmol/L, % of all BG	1.4 [0.0–2.8] (2.5)	1.4 [0.0–2.8] (2.0)	1.4 [0.0–5.6] (4.2)	0.31
Number of patients with BG <2.2 mmol/L	0	0	0	
BG measurements per day	15.1 [13.8–16.3]	15.1 [13.4–16.7]	15.4 [14.7–15.9]	0.66
Per-patient median insulin, U/h	3 [2–3]	3 [3–3]	3 [2–3]	0.40
Per-patient median feed, g/h	3.3 [1.9–4.8]	3.5 [1.9–4.6]	2.8 [2.1–5.6]	0.80

Data are given as median [IQR] unless otherwise indicated. *p* Values were computed using Fisher’s exact test and rank-sum tests where appropriate

*Abbreviations: APACHE* Acute Physiology and Chronic Health Evaluation, *BG* Blood glucose, *ICU* Intensive care unit, *SOFA* Sequential Organ Failure Assessment, *SPRINT* Specialised Relative Insulin Nutrition Tables protocol

^a^Equivalence, as explained in ‘Analyses and statistics’ subsection under ‘Methods’

### Model-based SI

**Fig. 2:**
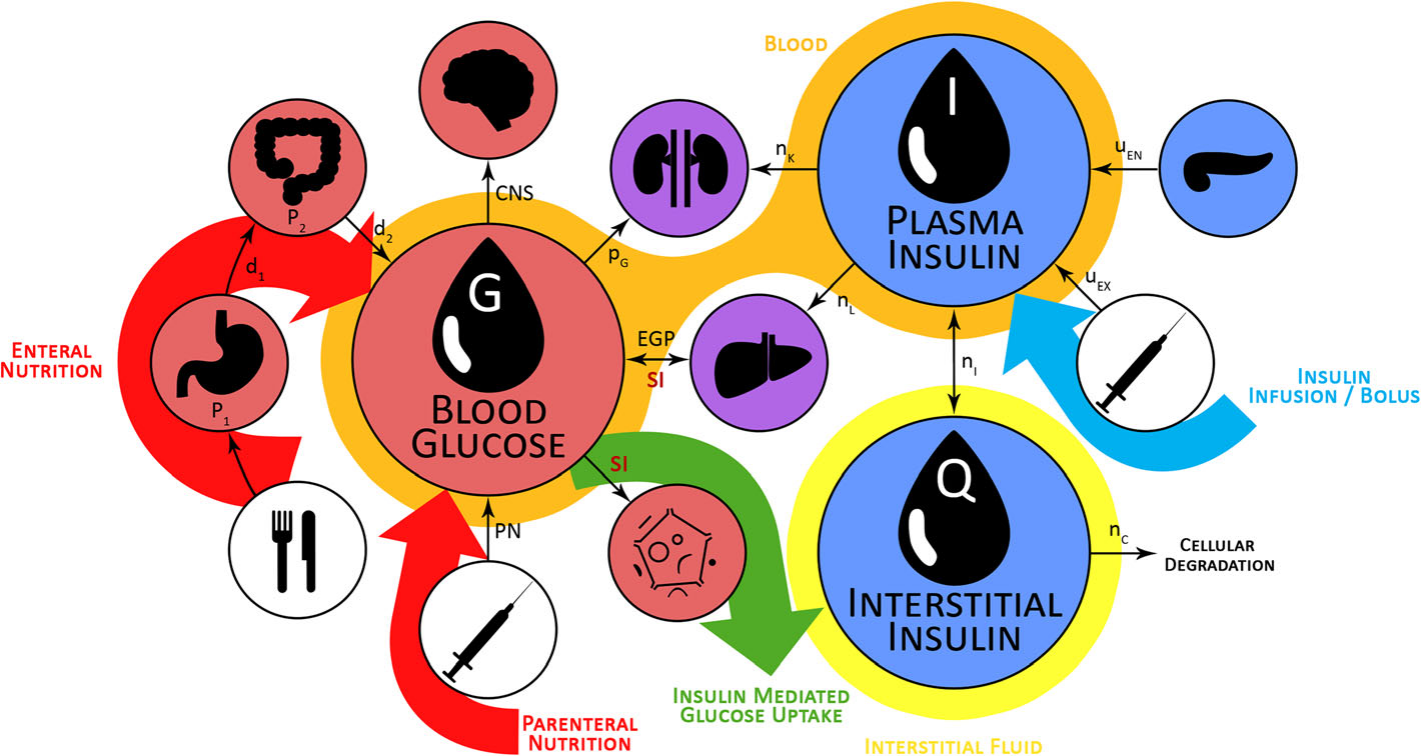
Schematic representation of the glucose-insulin model showing the physiological compartments and clearances, as well as the appearance of exogenous insulin and carbohydrate, and their kinetic pathways. *CNS* Central nervous system, *EGP* Endogenous glucose production, *PN* Parenteral nutrition, *SI* Insulin sensitivity

The physiological model-based glucose-insulin dynamics represented in Fig. 2 are defined by the following equations [49]:

1 $$ \overset{.}{G} = -{p}_G. G(t)-{S}_I. G(t)\frac{Q(t)}{1 + {\alpha}_G. Q(t)}+\frac{P(t)+ EGP- CNS}{V_G}, $$

2 $$ \overset{.}{I} = {n}_K. I(t)-{n}_L\frac{I(t)}{1 + {\alpha}_I. I(t)}-{n}_I\left( I(t)- Q(t)\right)+\frac{u_{ex}(t)}{V_I}+\left(1-{x}_L\right)\frac{u_{en}(G)}{V_I}, $$

3 $$ \overset{.}{Q} = {n}_I\left( I(t)- Q(t)\right)-{n}_C\frac{Q(t)}{1+{\alpha}_G Q(t)}. $$

The main model variables and parameters are described in Table 3, and the full model details and physiological relevance are presented in Additional file 1. SI is a patient-specific, time-varying parameter that characterises the systemic response to glucose and insulin administration. The SI units used (L/mU/minute) are consistent with a rate parameter for assessing the rate of insulin-mediated glucose removal, where litres per minute is a rate of flow or uptake and mU^−1^ makes it dependent on current insulin concentration. When SI is multiplied by the average hourly glucose for the period over which SI is calculated, the units become consistent with those used in the gold standard hyperinsulinaemic, euglycaemic clamp assessment of SI [51, 52]. Integral-based fitting [53] is used to determine SI hourly from clinical BG, insulin and nutrition-related data.

**Table 3:** Key variables of the Intensive Control Insulin-Nutrition-Glucose metabolic glucose model

Parameter	Description
G(t)	Blood glucose level (mmol/L)
I(t)	Plasma insulin concentration (mU/L)
Q(t)	Interstitial insulin concentration (mU/L)
P(t)	Glucose appearance in plasma from dextrose intake (mmol/minute)
*S*_*I*_	Insulin sensitivity (L/mU/minute)

Full table is provided in Additional file 1

SI level is determined hourly for each patient, and the forward SI variability (%ΔSI) is defined as the hour-to-hour percentage change in SI, calculated as follows:

$$ \%\varDelta S{I}_i=100 \times \frac{S{I}_{i+1} - S{I}_i}{S{I}_i} $$

A previous retrospective analysis showed that the SI of critically ill patients was lower and more variable during the first 24 h of the ICU stay, where SI was analysed in 6-h blocks [35, 54]. However, differences between survivors and non-survivors or other clinical outcomes were not analysed.

### Analyses and statistics

In this study, we analysed SI over the first 3 days (72 h) of GC and compared the evolution of SI and %ΔSI for survivors and non-survivors. Only patients who received insulin therapy under the SPRINT protocol during the first 12 h of ICU admission are included, so there are only small differences between ICU admission and time on the SPRINT protocol. This choice avoids any bias due to different time since ICU admission, given the evolution seen in previous studies [35, 36, 54] for the cohort as a whole.

SI and %ΔSI were analysed in 6-h blocks. Cumulative distribution functions (CDFs) for each metric were created for survivors and non-survivors over each 6-h block. These CDFs show the overall distribution and are defined exactly as the integral of the probability density function capturing the histogram of the data. Therefore, they clearly define the median and any percentile likelihood (*y*-axis) for any given SI or %ΔSI values (*x*-axis).

Hypothesis testing was used to examine differences, with *p* ≤ 0.05 used as a threshold for statistical significance. The Kolmogorov-Smirnov test was used to identify bias and shape difference in distributions of %ΔSI. Although it is not certain if each family of comparisons is strictly independent (i.e., each 6-h block may depend on surrounding blocks), for completeness and to be conservative, a Bonferroni correction for multiple comparisons was used to generalise the results. In both Cohorts 1 and 2, there were 12 comparisons made, bringing the significance level to *p* = 0.004 (0.05/12) [55].

Owing to a relatively large number of data points, bootstrapping was used to examine the difference between median SI and median %ΔSI between survivor and non-survivor cohorts [55]. Data were bootstrapped 1000 times with replacement to generate cohorts of the same size as the original data for a given 6-h block. A 95% CI for the difference between median SI values and between median %ΔSI values was generated. Where this CI does not cross zero, differences in medians are statistically significant with *p* ≤ 0.05 [55]. A 99.6% CI, consistent with using *p* = 0.004, was taken into account when considering Bonferroni correction for multiple comparisons.

Hypothesis testing was used to examine differences between cohorts and to assemble evidence to reject the null hypothesis of data being drawn from the same underlying distribution. However, it cannot provide evidence for equivalence, especially for large sample sizes [55–57]. Equivalence testing was used to assess the impact of these differences on clinical decision making, regardless of the underlying statistical significance (*p* value). Thus, it is important to note that a difference can be statistically significantly different and also equivalent, because the first is a statistical measure and the second is a measure of the clinical impact of the difference in the two distributions.

An analysis was done to determine an equivalence interval for changes in SI, as reflected by clinical significance. This interval thus defines the range within which a difference of medians cannot be distinguished, owing to either measurement error and/or clinical significance. Clinical significance was defined as the change in SI required to exceed BG measurement error (SD ±9.4% [58]) or to cause a change in model-based insulin dose recommendations. These calculations can be found in Additional file 2. In this case, the equivalence range due to measurement error was the narrowest across the range of clinical inputs observed. This choice provides the narrowest range and thus the most conservative or stringent test of equivalence.

The resulting equivalence range for %ΔSI is typically about 12–15%, but it is dependent on BG. Thus, any changes in SI or %ΔSI within these ranges cannot be detected as different from a change due to measurement error and are thus equivalent. Equivalence testing is independent of *p* values and hypothesis testing.

Equivalence was tested for SI and %ΔSI over each 6-h interval. For SI, the bootstrapped percentage difference in median SI was compared with the equivalence range. If the 95% CI for the bootstrapped percentage difference in SI medians was within the equivalence range, then equivalence in SI was accepted (⇔). For %ΔSI, the absolute difference in median %ΔSI was examined. If the 95% CI for the bootstrapped difference in median %ΔSI was within the equivalence range, then equivalence in %ΔSI was accepted (⇔). Conversely, in both cases, if the 95% CI was outside the equivalence range, equivalence was thus rejected (×). Finally, equivalence was tested for BG in Cohort 1 and Cohort 2 as a whole, using the reported equivalence range of ±9.4%, which is 1 SD of the relevant BG measurement error [58]. Equivalence testing in this last case determined whether the significant differences in median cohort BG in Tables 1 and 2 were clinically significant.

## Results

### SI level

**Fig. 3:**
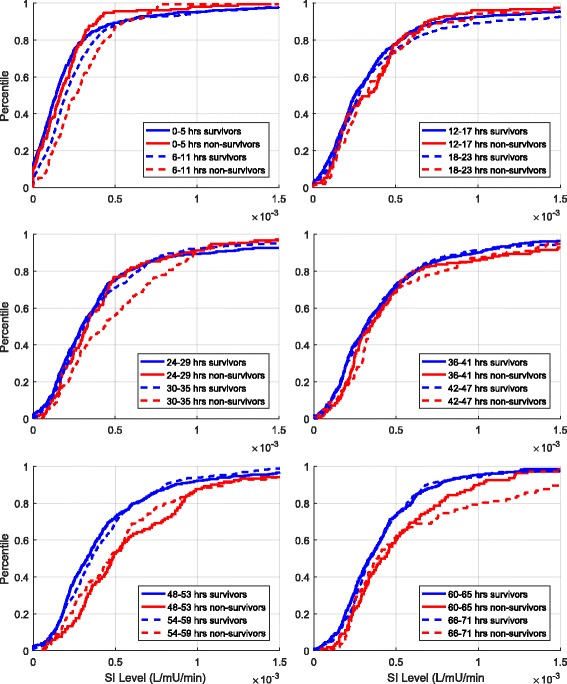
Cohort 1 cumulative insulin sensitivity (SI) levels over 6-h time intervals for the first 72 h of glycaemic control. At any level of SI, the *y*-axis gives the percentage of SI values (decimal percentile) below this level. The 95% CI on difference in medians was computed using bootstrapping

Table 4 shows median SI and IQR for survivors and non-survivors in both Cohort 1 and Cohort 2 over the first 72 h. The CDFs for SI over each 6 h block for Cohort 1 are shown in Fig. 3. Overall, SI level increases over time, matching [35], where non-survivors have higher SI than survivors.

**Table 4:** SI level (L/mU/minute) median [IQR] comparison between survivors and non-survivors using 6-h blocks

**Hours**	**Cohort 1**: (*n* = 145 patients)			
	Survivors (*SI*_*S*_) L/mU/minute × 10^−4^	Non-survivors (*SI*_*NS*_) L/mU/minute × 10^−4^	Median *SI*_*S*_ *− SI*_*NS*_ [95% CI] L/mU/minute × 10^−4^	
Day 1				
0–5	1.39 [0.50, 2.54]	1.64 [0.63, 2.63]	−0.25 [−0.60, 0.06]	**×**
6–11	1.94 [1.11, 3.35]	2.58 [1.42, 3.97]	**−0.63 [−1.04, −0.11]** ^a,b^	**×**
12–17	2.54 [1.42, 4.48]	3.39 [1.63, 4.79]	−0.79 [−1.46, 0.22]	**×**
18–23	2.76 [1.57, 5.09]	3.22 [1.93, 5.16]	−0.42 [−0.93, 0.14]	**×**
Day 2				
24–29	2.96 [1.65, 4.98]	3.30 [1.81, 4.85]	−0.30 [−0.73, 0.13]	**×**
30–35	3.08 [1.83, 5.73]	4.34 [2.35, 7.21]	**−1.23 [−2.16, −0.20]** ^a^	**×**
36–41	3.13 [1.81, 5.44]	3.42 [2.23, 5.36]	−0.29 [−1.01, 0.43]	**×**
42–47	3.22 [1.81, 5.47]	4.43 [2.48, 6.24]	−0.25 [−0.94, 0.16]	**×**
Day 3				
48–53	3.28 [1.95, 5.36]	4.83 [3.13, 8.63]	**−1.57 [−2.36, −0.97]** ^a,b^	**×**
54–59	3.55 [2.03, 5.50]	4.65 [2.53, 7.27]	**−1.12 [−2.04, −0.40]** ^a^	**×**
60–65	3.39 [2.18, 5.18]	4.19 [2.71, 6.83]	**−0.81 [−1.59, −0.01]** ^a^	**×**
66–71	3.40 [2.43, 5.07]	3.86 [2.43, 8.30]	−0.47 [−1.43, 0.16]	**×**
**Hours**	**Cohort 2**: (*n* = 80 patients)			
	Survivors (*SI*_*S*_) L/mU/minute × 10^−4^	Non − Survivors (*SI*_*NS*_) L/mU/minute × 10^−4^	Median *SI*_*S*_ *− SI*_*NS*_ [95% CI] L/mU/minute × 10^−4^	
Day 1				
0–5	1.39 [0.43, 2.45]	1.38 [0.30, 2.54]	−0.00 [−0.52, 0.57]	**×**
6–11	1.90 [0.92, 3.66]	2.22 [1.15, 3.62]	−0.33 [−1.00, 0.02]	**×**
12–17	2.36 [1.37, 4.48]	2.46 [1.46, 4.50]	−0.12 [−1.19, 0.61]	**×**
18–23	2.63 [1.53, 4.47]	2.94 [1.87, 4.50]	−0.30 [−0.81, 0.14]	**×**
Day 2				
24–29	2.95 [1.53, 4.52]	3.19 [1.65, 4.82]	−0.26 [−0.75, 0.22]	**×**
30–35	3.04 [1.88, 5.07]	3.56 [2.24, 6.85]	−0.55 [−1.95, 0.12]	**×**
36–41	3.06 [1.79, 4.94]	3.15 [2.14, 5.04]	−0.10 [−0.79, 0.51]	**×**
42–47	3.21 [1.80, 5.23]	3.41 [2.93, 5.27]	−0.24 [−0.86, 0.22]	**×**
Day 3				
48–53	3.31 [1.98, 5.30]	4.59 [3.03, 8.20]	**−1.26 [−1.84, −0.41]** ^a,b^	**×**
54–59	3.59 [2.09, 5.50]	4.37 [2.43, 7.36]	**−0.87 [−1.81, −0.09]** ^a^	**×**
60–65	3.45 [2.18, 5.24]	3.94 [2.62, 6.53]	−0.48 [−1.37, 0.25]	**×**
66–71	3.41 [2.43, 5.21]	3.68 [2.42, 7.56]	−0.30 [−1.20, 0.24]	**×**

Non-equivalence is indicated by ×, Equivalence is indicated by ⇔

^a^Hours where the medians are statistically different (95% CI on difference in medians does not cross zero)

^b^Differences remaining significant after Bonferroni correction (99.6% CI on difference in medians does not cross zero)

Bootstrapped confidence interval (CI) in bold is statistically significant to *p*<0.05

**Fig. 4:**
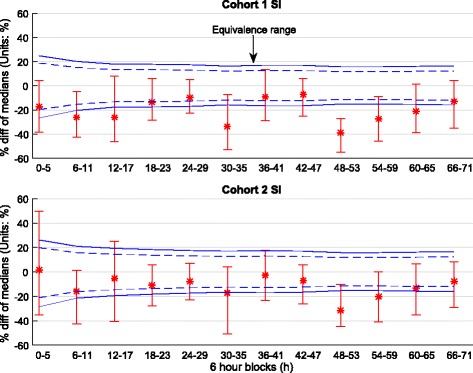
Equivalence testing on insulin sensitivity (SI) for each 6 h block for Cohort 1 and Cohort 2. The *solid blue lines* give equivalence ranges for 9.4% blood glucose error [58] and the *blue dotted lines* a smaller 7% error reported for the device used in highly controlled tests [105]. Equivalence is accepted if the 95% CI (*bars*) of bootstrapped percentage differences in median SI values is within the equivalence range, and rejected otherwise (×)

In Cohort 1, the difference between median SI levels was not statistically significant (95% CI crosses zero) for the first 48 h, except for 6–11 h and 30–35 h. By Day 3, the differences became significant, except for the 66–71 h block. With the Bonferroni correction applied, only the 6–11 h and 48–53 h blocks remained statistically different. In every 6-h block, non-survivors had higher SI levels than survivors. Figure 4 shows results of the equivalence test for each 6-h block. At no time did the median and 95% CI values for the percentage difference of SI medians in survivors and non-survivors fall within the equivalence range. Therefore, the median SI level was never equivalent in survivors and non-survivors, regardless of *p* values assessing difference.

The results were similar for Cohort 2. However, after Bonferroni correction, median SI was statistically different only for hours 48–53. Survivors and non-survivors were never equivalent, and SI was always higher for non-survivors in Cohort 2, all of whom had an ICU length of stay of 3 days or longer.

**Fig. 5:**
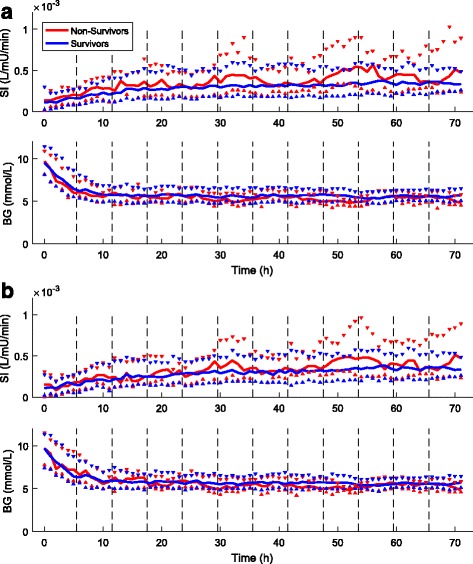
Median [IQR] evolution of insulin sensitivity (SI) and blood glucose (BG) over time for survivors (*blue*) and non-survivors (*red*) in Cohort 1 (**a**) and Cohort 2 (**b**)

Figure 5 shows the evolution of median [IQR] SI and BG values over time between survivors and non-survivors for Cohort 1 and Cohort 2. In both cohorts, SI was higher for non-survivors, as reflected in Table 4, and this difference was greater as control progressed. In terms of BG, survivors and non-survivors had similar levels for most hours. Equivalence testing on overall BG distributions between survivors and non-survivors showed the median and 95% CI of the percentage change in median BG were 5.3 [2.6, 7.1] for Cohort 1 and 3.5 [0.9, 5.3] for Cohort 2, which were well within equivalence ranges of 7.0–9.4%. Thus, whereas the differences are statistically different, they confirm that the differences in the median BG values in Tables 1 and 2 are not clinically significant. It is important to note that these two figures do not necessarily reflect SI hour-to-hour variability at a per-patient level. Two patients could have equal variability in a 6-h period but at different hours, and thus appear different in SI level, which explains the need for a separate %ΔSI analysis assessing the hour-to-hour variability.

### SI variability (%ΔSI)

**Fig. 6:**
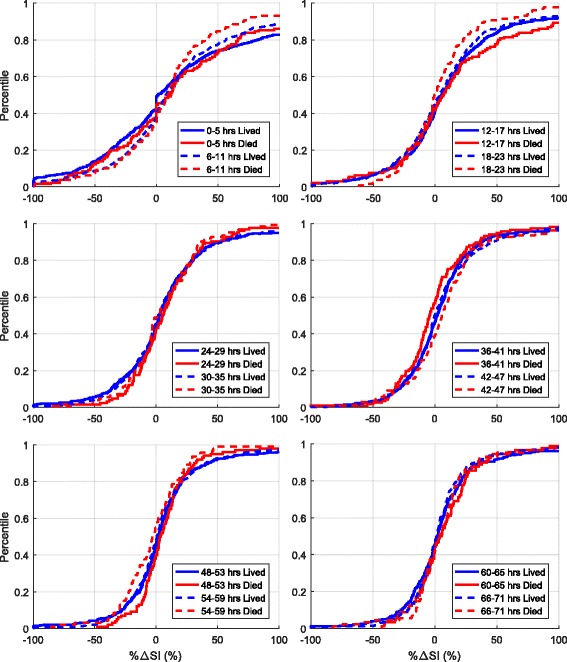
Cohort 1 cumulative hour-to-hour percentage changes in insulin sensitivity (%ΔSI) over 6-h time intervals for the first 72 h of glycaemic control. At any level of %ΔSI, the *y*-axis gives the percentage of %ΔSI values (decimal percentile) below this level. *p* Values were calculated using the Kolmogorov-Smirnov test

**Fig. 7:**
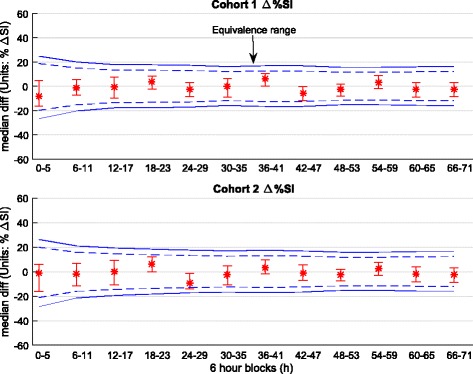
Equivalence testing on insulin sensitivity variability (hour-to-hour percentage change in insulin sensitivity [%ΔSI]) for each 6-h block for Cohort 1 and Cohort 2. The *solid lines* give equivalence ranges for 9.4% blood glucose error [58], and the *dotted lines* give a smaller 7% error reported for the device used in highly controlled tests [104]. Equivalence is accepted (⇔ in Table *5*) if the 95% CI (*bars*) of bootstrapped difference in median %ΔSI are within the equivalence range, and rejected otherwise (×)

Results for %ΔSI are shown in Table 5 and Fig. 6. Overall, SI variability decreased over time (IQR narrows) for both survivors and non-survivors, matching previously reported results [35]. In both Cohort 1 and Cohort 2, %ΔSI was not significantly different (*p* ≥ 0.11 in 11 of 12 blocks), especially when the Bonferroni correction for multiple comparisons was made (*p* < 0.004 correction threshold). The 95% CI on median difference in %ΔSI (bias only) can be considered significant only for the 36–41 h and 42–47 h blocks in Cohort 1 and only for the 18–23 and 24–29 h blocks in Cohort 2 (bootstrapping, right-most column of Table 5), but these significances did not hold when the Bonferroni correction was made (99.6% CI). In all cases, these differences were not clinically significant. As shown in Fig. 7, the median and 95% CI change in %ΔSI difference was always within the equivalence range for both Cohorts 1 and 2. Therefore, SI variability assessed as %ΔSI in survivors and non-survivors was equivalent in every 6-h block to 72 h.

**Table 5:** Hour-to-hour percentage change in insulin sensitivity (%ΔSI) ﻿media﻿n [IQR] comparison between survivors and non-survivors using 6-h blocks

**Hours**	**Cohort 1**: (*n* = 145 patients)				
	Survivors (%∆*SI*_*S*_) %	Non-survivors (%∆*SI*_*NS*_) %	Kolmogorov-Smirnov test *p* value	Median %∆*SI*_*S*_-%∆*SI*_*NS*_ [95% CI] %	
Day 1					
0–5	1.46 [−29.26, 54.74]	11.67 [−20.84, 56.41]	0.67	−8.12 [−16.22, 4.67]	⇔
6–11	7.37 [−14.66, 42.05]	9.47 [−11.45, 27.98]	0.53	−1.31 [−6.22, 5.74]	⇔
12–17	5.21 [−11.87, 30.89]	6.69 [−14.89, 42.15]	0.62	−0.98 [−9.46, 7.26]	⇔
18–23	3.24 [−16.02, 26.92]	−0.63 [−12.21, 16.37]	0.12	3.72 [−1.99, 8.56]	⇔
Day 2					
24–29	2.79 [−13.36, 23.35]	5.37 [−9.42, 23.52]	0.30	−2.70 [−8.60, 3.29]	⇔
30–35	1.76 [−15.13, 23.46]	1.57 [−11.32, 24.75]	0.78	0.34 [−8.54, 6.75]	⇔
36–41	1.92 [−12.19, 16.87]	−4.01 [−15.63, 11.26]	**0.04**	**6.10 [0.35, 10.70]** ^a^	⇔
42–47	−0.10 [−12.71, 17.98]	5.46 [−10.91, 21.91]	0.14	**−5.66 [−11.61, −0.43]** ^a^	⇔
Day 3					
48–53	1.57 [−10.74, 16.82]	3.41 [−7.30, 14.99]	0.30	−2.12 [−7.41, 1.77]	⇔
54–59	0.67 [−11.68, 15.80]	−3.13 [−19.08, 11.65]	0.35	3.37 [−1.77, 8.20]	⇔
60–65	2.39 [−12.39, 17.03]	4.89 [−8.88, 21.88]	0.45	−2.50 [−9.06, 3.35]	⇔
66–71	1.26 [−9.80, 12.87]	3.78 [−8.82, 15.48]	0.35	−2.76 [−8.66, 2.80]	⇔
**Hours**	**Cohort 2**: (*n* = 80 patients)				
	Survivors (%∆*SI*_*S*_) %	Non-survivors (%∆*SI*_*NS*_) %	Kolmogorov-Smirnov test *p* value	Median %∆*SI*_*S*_-%∆*SI*_*NS*_ [95% CI] %	
Day 1					
0–5	0 [−29.44, 43.57]	0.98 [−20.90, 57.81]	0.78	−0.98 [−16.02, 5.93]	⇔
6–11	8.80 [−14.66, 48.55]	10.59 [−17.24, 39.20]	0.90	−2.17 [−11.46, 6.83]	⇔
12–17	2.38 [−13.18, 29.19]	2.92 [−15.99, 38.92]	0.89	−0.02 [−11.00, 9.19]	⇔
18–23	4.09 [−14.80, 26.14]	−2.13 [−11.66, 15.29]	0.11	**6.16 [0.10, 12.10]** ^a^	⇔
Day 2					
24–29	1.32 [−13.48, 20.79]	10.39 [−8.97, 25.86]	**0.02**	**−9.23 [−14.18, −1.38]** ^a^	⇔
30–35	0.13 [−15.56, 21.72]	3.08 [−13.33, 23.03]	0.89	−2.38 [−10.72, 4.79]	⇔
36–41	2.54 [−12.13, 18.20]	0.40 [−11.95, 15.13]	0.39	2.95 [−1.86, 9.51]	⇔
42–47	1.37 [−13.37, 22.76]	2.42 [−11.83, 14.68]	0.46	−1.02 [−7.15, 5.68]	⇔
Day 3					
48–53	0.88 [−10.32, 16.63]	3.16 [−7.25, 14.62]	0.30	−2.37 [−7.73, 1.79]	⇔
54–59	0.72 [−10.36, 14.28]	−1.17 [−19.08, 12.68]	0.32	2.69 [−3.18, 7.71]	⇔
60–65	2.58 [−10.54, 16.38]	4.04 [−9.03, 21.96]	0.39	−1.89 [−8.44, 3.76]	⇔
66–71	1.26 [−9.74, 11.54]	3.72 [−9.06, 14.81]	0.40	−2.62 [−8.81, 3.12]	⇔

Equivalence is indicated by ⇔, Non-equivalence is indicated by ×

^a^Hours where the medians are statistically different (95% CI on difference in medians does not cross zero)

Bootstrapped confidence interval (CI) in bold is statistically significant to *p*<0.05

### Key results

In summary, the key results are as follows:

- SI level is not equivalent in any 6-h block within the first 72 h of GC, and it is sometimes statistically different between survivors and non-survivors.
- SI level is higher in non-survivors than in survivors in every 6-h block for the first 72 h, and this difference becomes statistically significant as GC progresses.
- SI variability is equivalent between survivors and non-survivors in any 6-h block within the first 72 h of GC.
- Patient dropout had no impact on results, because Cohort 2 had the same key outcomes.
- The major results are consistent, regardless of whether the Bonferroni correction for multiple comparisons is applied.

Thus, whereas survivors and non-survivors differed in their absolute SI, with non-survivors having higher SI, they were equivalent in their %ΔSI.

## Discussion

### Primary question

Patient-specific SI and SI variability metrics are used to assess underlying controllability between survivors and non-survivors. Both statistical difference and equivalence were tested in comparing these cohorts. Statistical difference (*p* < 0.05) tests whether the data come from similar or different distributions, whereas, in contrast, equivalence tests whether these values are clinically or physiologically equivalent, regardless of *p* value.

SI was statistically different between survivors and non-survivors for five of twelve 6-h periods. However, the cohorts were never clinically equivalent in SI for any period. Non-survivors had higher SI in every period, suggesting that slightly lower insulin doses would be required to achieve normo-glycaemia, which is also seen in the clinical results in Table 1. Key results were the same for both cohorts examined.

%ΔSI was equivalent between survivors and non-survivors for every period, and it was statistically different in only two periods. Equivalent variability under the same GC protocol would be reflected in similar times in glycaemic bands and in glycaemic levels for both survivors and non-survivors, as seen in Table 1. The results were the same for both cohorts. Median BG was higher in survivors (5.5 vs 5.8 mmol/L, *p* < 0.01 for both cohorts), but this difference was shown to be clinically equivalent in terms of measurement error and, in addition, would not change the clinical interventions.

Whereas SI level tends to determine the total insulin dose titrated, it is variability that determines the risks of insulin therapy and overall controllability. Overall, similar to higher SI for non-survivors and equivalent variability suggest that survivors and non-survivors are equally controllable, given an effective GC protocol. This outcome in turn suggests that the association between glycaemia and outcome is thus predominated by the quality of GC achieved and not by underlying patient variability. This result is important and has implications for GC study design and practice.

### Validity of SI metric

The results rely on the validity of the model-based estimate of SI. The reliability of the SI metric is determined by the underlying data and the ability of the model to capture key glucose-insulin dynamics. The Intensive Control Insulin-Nutrition-Glucose (ICING) model used in the present study is structurally very similar to the Dynamic Insulin Sensitivity and Secretion Test model, for which the SI metric has correlated well with the gold standard euglycaemic clamp SI values [59–62], as have other very similar models using the SI metrics and pharmacodynamics used here [52]. The ICING model and its SI metric have been used successfully and safely to guide insulin therapy across different adult [47, 48, 63, 64] and neonatal [65, 66] intensive care settings and delivery methods. These clinical results suggest that the model is able of capturing and accounting for all major glucose-insulin dynamics, making the SI parameter, and its guiding of care via forward prediction, clinically useful.

In addition, treatment independence of the SI parameter has been assessed using clinical data from independent, matched patient cohorts [46, 67]. In the first case, two cohorts and protocols (Glucontrol [14] and SPRINT [10]) from Liège, Belgium, and Christchurch, New Zealand, were simulated with both protocols, and their glycaemic level and variability were compared with those obtained clinically. Consistency in simulation results across cohorts and high similarity in stochastic plots of SI variability further validated the treatment and cohort independence of SI [68]. In the second case, this similarity and cross-validation were repeated across three medical ICU cohorts, further validating these outcomes [67]. Recent work suggests that it is an underlying similarity in SI variability, independent of absolute SI level, that drives GC outcomes [46, 67, 68]. This similarity thus also drove the observed consistency between clinical results using this model and SI metric for GC in two very different ICUs [64].

Moreover, SI has been shown to assess and reflect clinically expected changes in SI and metabolism for important intensive care interventions. The impact of glucocorticoids [69] and β-blockers [70] on SI level and %ΔSI was shown to be limited in the context of the SPRINT protocol. More specifically, insulin and nutrition inputs were not statistically different in this study between survivors and non-survivors (*p* > 0.34) (Table 1), where increasing insulin use would reflect increased insulin resistance (lower SI). These results thus suggest that glucocorticoid-mediated influence on SI does not have any net impact on the two groups, as there was such a difference in the study by Pretty et al. [69]. Additionally, the impact of exogenous nutrition and incretin effects seen in changes in SI [71], the impact on SI from haemodialysis altering insulin clearance [72], and finally the insulin resistance observed on and off therapeutic hypothermia [73] were all assessed using hourly identified SI based on the same model. Each of these studies demonstrated the ability of SI and its changes to reflect clinically expected outcomes and correlated with expectations for the given intervention.

Other factors, such as insulin administration form (bolus vs continuous dosing), have little impact on the hourly calculated SI value. In this study, both survivors and non-survivors were treated with bolus doses, eliminating any effect that could exist for this comparison. Glucose sensor errors could have a more measurable impact on SI calculation [74], but the same glucometers were used for all patients, similarly ameliorating this affect. Continuous glucose monitoring (CGM) delivers observations indicating greater apparent spontaneous variability in BG levels than seen with typical intermittent sampling. However, it is important to note that a major part of this CGM-observed BG variability is due not to patient metabolism but directly to sensor drift, changes in the in situ environment of the sensor, patient position and other factors [75–82]. Thus, what is captured by CGM may be either realistic or an artefact or some combination. However, differentiating these systemic errors from real BG variability is not currently possible without another reference measurement at the same rate. As a result, the hourly determined SI values used here are appropriate, particularly with regard to the measurement rate in the data, which cannot capture any real glycaemic variability in the data that occurs and resolves between measurements. Hence, the overall approach used here is appropriate to the data and its sampling rate and does captures very high levels of variability, as seen in Fig. 6 with changes in SI up to 640%. Two examples of SI profiles over time, indicating the actual variability possible, are shown in Additional file 1.

Glucose complexity has been associated with mortality [83, 84] but cannot be measured at the bedside in real time as glycaemic levels, time in band, or variability can. Equally, there is not the strong physiological evidence that would support this association which exists for the other metrics considered, and there are questions about its proper use in analysing continuous glucose data to create these associations [85, 86].

The presented results suggest non-survivors have higher SI, which at first appeared counter-intuitive. However, it can be hypothesised that some non-survivors may have had weaker inflammatory immune responses and/or weaker inflammatory counter-regulatory response to insult. Although the literature commonly points to increased inflammatory markers in non-survivors (e.g., [87, 88]), there is evidence of instances where compromised immune response leads to increased mortality (e.g., [89–92]). These physiological responses (both inflammatory [93–98] and counter-regulatory [94, 99–102]) drive hyperglycaemia via the inflammatory marker-induced actions that reduced the effective SI values analysed here. They are also two of three major drivers of hyperglycaemia, the third being high glucose itself. Hence, weakened responses in those who die would lead to slightly higher SI and thus may be the cause of the slightly higher SI and slightly lower, clinically speaking, insulin use in this cohort. We do not have evidence to prove this hypothesis, but it would make a good hypothesis for a future study.

In particular, SI is approximately 20% higher, on average, for non-survivors, ranging from about 9–40% over time periods, which is at or within the level of change in SI required to induce, in SPRINT, a 1-U/h change in insulin dose, considering a median of 3 U/h (see Additional file 2: Figure S2.5). Thus, this difference changed few interventions, as seen in Table 1 (median [IQR] of 3 [2, 3] U/h for both survivors and non-survivors), where feed is also similar. Excluding dropout in Cohort 2, the differences remained but were much smaller (approximately 12%). Thus, although SI is higher for non-survivors and not equivalent to SI of survivors, on the basis of the most conservative estimate (percentage change in SI to reach 9.4% BG measurement error), this difference in SI did not have a significant clinical impact in terms of interventions, where an approximately 20–25% change in SI was required to change an intervention (see Additional file 2: Figure S2.5).

One advantage of the model-based SI used here is that it accounts for all insulin and nutrition inputs as well as resulting changes in glycaemia, allowing the SI metric to reflect the underlying ability of the body to use insulin for glucose uptake. Using SI thus allows an objective numerical analysis to be carried out and for results to be generalised to other mixed ICU populations.

### Advantages and limitations

A first potential limitation of this work is that, as with all models, the ICING model has ranges for BG and nutrition-insulin interventions in which it is most accurate [103]. These ranges span what is typically observed in the Christchurch Hospital ICU, including BG within the 4–10 mmol/L range and insulin and nutrition treatments within 0–10 U/h and 20–120% of goal feed, respectively. If this analysis were to be repeated in ICUs or with protocols where treatments may commonly be given outside these ranges, or where persistent hyper- and/or hypoglycaemia were common, there would be greater potential for analytical error. However, in this case, the clinical data and inputs all fall within the ideal range for the ICING model.

A significant advantage of this work is that it uses data of sufficient detail and quality for further analysis. Many studies do not record (or report) detailed nutrition and/or insulin inputs, so analyses are limited by either disregarding nutrition in the first place or considering daily averages and effects. This data set included all time-valued changes in insulin and nutrition in 1-2 h intervals, as well as all BG measures, thus allowing a much higher degree of resolution in the calculation of time-varying SI. The limitation is that this analysis would be difficult to repeat with data from other, larger studies because of this lack of detail and/or temporal resolution of the GC data collected.

This study is limited in its retrospective nature and because it was performed with data from a single centre. However, the data cover a relatively large, generalised patient cohort spanning several years of clinical practice. Illness and injury can affect the inflammatory response and thus the SI. The analysis cohorts were therefore selected on the basis of starting GC within the first 12 h of ICU stay to reduce the effect of time-varying degrees of illness and injury on the time-varying analysis of SI.

## Conclusions

The results we report show equivalent metabolic variability between survivors and non-survivors and that non-survivors had higher SI. These results are based on a numerical, objective, model-based SI metric which takes into consideration both nutrition-insulin inputs and metabolic outcomes. The underlying data cohort is derived from a mixed medical ICU, and as previous work has shown consistency in variability across different cohorts, countries and centres, it is likely that the results of this study are not specific to the original data set. Overall, these results suggest that glycaemic outcomes and differences between survivors and non-survivors are thus more a function of the control provided rather than the underlying metabolic condition.

This outcome has implications for future study and protocol design in this area. Future work is required to confirm these results and explore the relationship between outcomes and GC.

## Additional files

Additional file 1: Metabolic system model and insulin sensitivity. This file presents additional details on the physiological model and methods used to calculate a patient’s time-varying insulin sensitivity used in this study. (DOCX 375 kb)

Additional file 2: Clinical significance calculations. This file presents the underlying calculations used to define the clinical ‘equivalence range’ used in this study. (DOCX 68 kb)

## Abbreviations

%ΔSI: Hour-to-hour percentage change in insulin sensitivity
APACHE: Acute Physiology and Chronic Health Evaluation
BG: Blood glucose
CDF: Cumulative distribution function
CGM: Continuous glucose monitoring
CNS: Central nervous system
EGP: Endogenous glucose production
GC: Glycaemic control
ICING: Intensive Control Insulin-Nutrition-Glucose model
ICU: Intensive care unit
PN: Parenteral nutrition
SI: Insulin sensitivity
SOFA: Sequential Organ Failure Assessment
SPRINT: Specialised Relative Insulin Nutrition Tables protocol
