# Supplementary material for: Untangling glycaemia and mortality in critical care
Source: Crit Care. 2017 Jun 24;21:152. doi: 10.1186/s13054-017-1725-y (PMC5482947; doi:10.1186/s13054-017-1725-y)
Supplement: Supplementary file 1 — Metabolic system model and insulin sensitivity. This file presents additional details on the physiological model and methods used to calculate a patient’s time-varying insulin sensitivity used in this study. (DOCX 375 kb) [file 13054_2017_1725_MOESM1_ESM.docx]

**Additional File 1: Metabolic System Model and Insulin Sensitivity (SI)**:

This Additional File is designed to present the model and methods used in several referenced studies (e.g. [[1-9](#_ENREF_1)]) in this paper. The presentation is brief, relying on a separate set of references (from the main article) given at the end of this Additional File, which interested readers can use for explicit details on any aspect of this model and the methods used.

A1-1 Model Definition:

A clinically validated computer model of the metabolic system [[10](#_ENREF_10)] was used to identify [[11](#_ENREF_11)] patient-specific, time-varying (hourly) insulin sensitivity (SI) every hour. The model presented is a compartment model, accounting for the appearance of insulin and glucose in blood and interstitial fluid volumes. Figure A1-1 shows this model (Figure 1 in the paper) schematically.

| $\dot{G}\left( t \right)=-p_{G}G\left( t \right)-S_{I}G\left( t \right)\frac{Q\left( t \right)}{1+\alpha_{G}Q\left( t \right)}+\frac{P\left( t \right)+EGP-CNS}{V_{G}}$ | 1 |
| --- | --- |
| $\dot{Q}(t)=n_{I}\left( I\left( t \right)-Q\left( t \right) \right)-n_{C}\frac{Q\left( t \right)}{1+\alpha_{G}Q\left( t \right)}$ | 2 |
| $\dot{I}\left( t \right)=n_{K}I\left( t \right)-n_{L}\frac{I\left( t \right)}{1+\alpha_{I}I\left( t \right)}-n_{I}\left( I\left( t \right)-Q\left( t \right) \right)+\frac{u_{ex}\left( t \right)}{V_{I}}+\left( 1-x_{L} \right)\frac{u_{en}(G)}{V_{I}}$ | 3 |
| $P\left( t \right)=\min\left( d_{2}P2, P_{\max} \right)+PN(t)$ | 4 |
| $\dot{P1}\left( t \right)=-d_{1}P1+D(t)$ | 5 |
| $\dot{P2}\left( t \right)=-min \left( d_{2}P2, P_{\max} \right)+d_{1}P1$ | 6 |
| $u_{en}\left( G \right)={min(max(u_{min}, k}_{1}G\left( t \right)+k_{2}), u_{max})$ | 7 |

Where *G(t)* [mmol/L] is plasma glucose concentration, *I(t)* and *Q(t)* [mU/L] are plasma and interstitial insulin concentrations. Pancreatic insulin secretion is modelled as a function of plasma glucose and is denoted *u_en_(G).* The associated parameter values and descriptions are listed in Table A1-1. Table A1-2 shows the exogenous input variables to the model.

**Table A1-1**. Parameter values and descriptions for the glucose-insulin model. Abbreviations;

|  | Value | Description | Fixed? | Identification Method | identification data set | Reported range |
| --- | --- | --- | --- | --- | --- | --- |
| *S_I_(t)* | l/mU/min | Insulin sensitivity | N | Integral based fitting [[1](file:///C:\Users\jld88\AppData\Local\Microsoft\Windows\Temporary%20Internet%20Files\Content.Outlook\6WEZMPQW\Appendix_on_model.docx#_ENREF_1)] | Clinical glucose, insulin, nutrition data | - |
| $\alpha_{G}$ | 1/65 (0.015) l/mU | Saturation of insulin-mediated glucose uptake | Y | Chosen from literature [[2](file:///C:\Users\jld88\AppData\Local\Microsoft\Windows\Temporary%20Internet%20Files\Content.Outlook\6WEZMPQW\Appendix_on_model.docx#_ENREF_2)]. Sensitivity tested in [[3](file:///C:\Users\jld88\AppData\Local\Microsoft\Windows\Temporary%20Internet%20Files\Content.Outlook\6WEZMPQW\Appendix_on_model.docx#_ENREF_3)]. | Literature review: [[4-7](file:///C:\Users\jld88\AppData\Local\Microsoft\Windows\Temporary%20Internet%20Files\Content.Outlook\6WEZMPQW\Appendix_on_model.docx#_ENREF_4)] | 0.001 – 0.025 l/min [[8](file:///C:\Users\jld88\AppData\Local\Microsoft\Windows\Temporary%20Internet%20Files\Content.Outlook\6WEZMPQW\Appendix_on_model.docx#_ENREF_8)]. |
| $p_{G}$ | 0.006 min^-1^ | Other non-insulin mediated glucose clearance | Y | Identified: grid search and error minimisation [[9](file:///C:\Users\jld88\AppData\Local\Microsoft\Windows\Temporary%20Internet%20Files\Content.Outlook\6WEZMPQW\Appendix_on_model.docx#_ENREF_9)] | Grid search: SPRINT cohort [[10](file:///C:\Users\jld88\AppData\Local\Microsoft\Windows\Temporary%20Internet%20Files\Content.Outlook\6WEZMPQW\Appendix_on_model.docx#_ENREF_10)]  Literature review: [[11-14](file:///C:\Users\jld88\AppData\Local\Microsoft\Windows\Temporary%20Internet%20Files\Content.Outlook\6WEZMPQW\Appendix_on_model.docx#_ENREF_11)] | 0.004 – 0.047 min^-1^ [[9](file:///C:\Users\jld88\AppData\Local\Microsoft\Windows\Temporary%20Internet%20Files\Content.Outlook\6WEZMPQW\Appendix_on_model.docx#_ENREF_9)] |
| $V_{G}$ | 13.3 L | Glucose distribution volume | Y | Chosen from literature [[9](file:///C:\Users\jld88\AppData\Local\Microsoft\Windows\Temporary%20Internet%20Files\Content.Outlook\6WEZMPQW\Appendix_on_model.docx#_ENREF_9)] | - | 10.0 – 15.75 L [[15](file:///C:\Users\jld88\AppData\Local\Microsoft\Windows\Temporary%20Internet%20Files\Content.Outlook\6WEZMPQW\Appendix_on_model.docx#_ENREF_15)]  0.22 L/kg [[16](file:///C:\Users\jld88\AppData\Local\Microsoft\Windows\Temporary%20Internet%20Files\Content.Outlook\6WEZMPQW\Appendix_on_model.docx#_ENREF_16)] |
| $EGP$ | 1.16 mmol/min | Endogenous glucose production (hepatic) | Y | Grid search and error minimisation [[9](file:///C:\Users\jld88\AppData\Local\Microsoft\Windows\Temporary%20Internet%20Files\Content.Outlook\6WEZMPQW\Appendix_on_model.docx#_ENREF_9)]. Later (unsuccessful) analysis as function of glucose and time [[17](file:///C:\Users\jld88\AppData\Local\Microsoft\Windows\Temporary%20Internet%20Files\Content.Outlook\6WEZMPQW\Appendix_on_model.docx#_ENREF_17)]. | Grid search and functional analysis: SPRINT cohort [[10](file:///C:\Users\jld88\AppData\Local\Microsoft\Windows\Temporary%20Internet%20Files\Content.Outlook\6WEZMPQW\Appendix_on_model.docx#_ENREF_10)]  Literature review (critically ill patients): [[18-28](file:///C:\Users\jld88\AppData\Local\Microsoft\Windows\Temporary%20Internet%20Files\Content.Outlook\6WEZMPQW\Appendix_on_model.docx#_ENREF_18)] | 0.10 – 2.36 mmol/min [[17](file:///C:\Users\jld88\AppData\Local\Microsoft\Windows\Temporary%20Internet%20Files\Content.Outlook\6WEZMPQW\Appendix_on_model.docx#_ENREF_17)]. |
| $CNS$ | 0.3 mmol/min | Glucose uptake by central nervous system | Y | Chosen from literature [[9](file:///C:\Users\jld88\AppData\Local\Microsoft\Windows\Temporary%20Internet%20Files\Content.Outlook\6WEZMPQW\Appendix_on_model.docx#_ENREF_9)]. | Literature review: [[29-35](file:///C:\Users\jld88\AppData\Local\Microsoft\Windows\Temporary%20Internet%20Files\Content.Outlook\6WEZMPQW\Appendix_on_model.docx#_ENREF_29)] | 0.29 – 0.38 mmol/min [[9](file:///C:\Users\jld88\AppData\Local\Microsoft\Windows\Temporary%20Internet%20Files\Content.Outlook\6WEZMPQW\Appendix_on_model.docx#_ENREF_9)]. |
| $x_{L}$ | 0.67 | Fractional first pass hepatics insulin clearance from portal vein | Y | Chosen from literature [[9](file:///C:\Users\jld88\AppData\Local\Microsoft\Windows\Temporary%20Internet%20Files\Content.Outlook\6WEZMPQW\Appendix_on_model.docx#_ENREF_9)] | Literature review: [[36-38](file:///C:\Users\jld88\AppData\Local\Microsoft\Windows\Temporary%20Internet%20Files\Content.Outlook\6WEZMPQW\Appendix_on_model.docx#_ENREF_36)] | 0.5-0.95 [[39](file:///C:\Users\jld88\AppData\Local\Microsoft\Windows\Temporary%20Internet%20Files\Content.Outlook\6WEZMPQW\Appendix_on_model.docx#_ENREF_39)]. |
| $n_{L}$ | 0.1578 min^-1^ | Rate parameter: general hepatic insulin clearance | Y | Chosen based on previous work [[9](file:///C:\Users\jld88\AppData\Local\Microsoft\Windows\Temporary%20Internet%20Files\Content.Outlook\6WEZMPQW\Appendix_on_model.docx#_ENREF_9)] | Normoglycaemic insulin resistant clamp study participants. [[15](file:///C:\Users\jld88\AppData\Local\Microsoft\Windows\Temporary%20Internet%20Files\Content.Outlook\6WEZMPQW\Appendix_on_model.docx#_ENREF_15), [40](file:///C:\Users\jld88\AppData\Local\Microsoft\Windows\Temporary%20Internet%20Files\Content.Outlook\6WEZMPQW\Appendix_on_model.docx#_ENREF_40)] | 0.1 – 0.21 min^-1^ [[15](file:///C:\Users\jld88\AppData\Local\Microsoft\Windows\Temporary%20Internet%20Files\Content.Outlook\6WEZMPQW\Appendix_on_model.docx#_ENREF_15)] |
| $\alpha_{I}$ | 1.7x10^-3^ l/mU | Saturation of hepatics insulin clearance | Y | Chosen from literature [[41](file:///C:\Users\jld88\AppData\Local\Microsoft\Windows\Temporary%20Internet%20Files\Content.Outlook\6WEZMPQW\Appendix_on_model.docx#_ENREF_41)]. | Literature review: [[42-47](file:///C:\Users\jld88\AppData\Local\Microsoft\Windows\Temporary%20Internet%20Files\Content.Outlook\6WEZMPQW\Appendix_on_model.docx#_ENREF_42)] | 0.0005 – 0.0043 L/mU [[8](file:///C:\Users\jld88\AppData\Local\Microsoft\Windows\Temporary%20Internet%20Files\Content.Outlook\6WEZMPQW\Appendix_on_model.docx#_ENREF_8)]. |
| $n_{K}$ | 0.0542 min^-1^ | Rate parameter: kidney clearance of insulin | Y | Chosen from literature [[9](file:///C:\Users\jld88\AppData\Local\Microsoft\Windows\Temporary%20Internet%20Files\Content.Outlook\6WEZMPQW\Appendix_on_model.docx#_ENREF_9)]. | - | 0.053–0.064 min^-1^[[15](file:///C:\Users\jld88\AppData\Local\Microsoft\Windows\Temporary%20Internet%20Files\Content.Outlook\6WEZMPQW\Appendix_on_model.docx#_ENREF_15)]. |
| $n_{C}$ | 0.006 min^-1^ | Rate parameter: cellular degradation of internalised insulin | Y | Identified: grid search and error minimisation [[3](file:///C:\Users\jld88\AppData\Local\Microsoft\Windows\Temporary%20Internet%20Files\Content.Outlook\6WEZMPQW\Appendix_on_model.docx#_ENREF_3)] | Published microdialysis studies [[48-53](file:///C:\Users\jld88\AppData\Local\Microsoft\Windows\Temporary%20Internet%20Files\Content.Outlook\6WEZMPQW\Appendix_on_model.docx#_ENREF_48)] | Parameter sensitivity: [[3](file:///C:\Users\jld88\AppData\Local\Microsoft\Windows\Temporary%20Internet%20Files\Content.Outlook\6WEZMPQW\Appendix_on_model.docx#_ENREF_3)]. |
| $n_{I}$ | 0.006 min^-1^ | Rate parameter: diffusion of insulin between plasma and interstitium | Y | Identified: grid search and error minimisation [[3](file:///C:\Users\jld88\AppData\Local\Microsoft\Windows\Temporary%20Internet%20Files\Content.Outlook\6WEZMPQW\Appendix_on_model.docx#_ENREF_3)] | Published microdialysis studies [[48-53](file:///C:\Users\jld88\AppData\Local\Microsoft\Windows\Temporary%20Internet%20Files\Content.Outlook\6WEZMPQW\Appendix_on_model.docx#_ENREF_48)] | 0 – 0.06 min^-1^ [[3](file:///C:\Users\jld88\AppData\Local\Microsoft\Windows\Temporary%20Internet%20Files\Content.Outlook\6WEZMPQW\Appendix_on_model.docx#_ENREF_3)]. |
| $k_{1}$ | 14.9 mU·l/mmol/min | Insulin secretion model parameter | Y | Model fit to clinical C-peptide and Insulin data. Compared to results derived from literature [[17](file:///C:\Users\jld88\AppData\Local\Microsoft\Windows\Temporary%20Internet%20Files\Content.Outlook\6WEZMPQW\Appendix_on_model.docx#_ENREF_17)]. | Clinical sepsis study patients [[17](file:///C:\Users\jld88\AppData\Local\Microsoft\Windows\Temporary%20Internet%20Files\Content.Outlook\6WEZMPQW\Appendix_on_model.docx#_ENREF_17)].  Literature review: [[54-61](file:///C:\Users\jld88\AppData\Local\Microsoft\Windows\Temporary%20Internet%20Files\Content.Outlook\6WEZMPQW\Appendix_on_model.docx#_ENREF_54)] | 8 - 45.9 mU/min [[17](file:///C:\Users\jld88\AppData\Local\Microsoft\Windows\Temporary%20Internet%20Files\Content.Outlook\6WEZMPQW\Appendix_on_model.docx#_ENREF_17)]. |
| $k_{2}$ | -49.9 mU/min | Insulin secretion model parameter | Y | Model fit to clinical C-peptide and Insulin data [[17](file:///C:\Users\jld88\AppData\Local\Microsoft\Windows\Temporary%20Internet%20Files\Content.Outlook\6WEZMPQW\Appendix_on_model.docx#_ENREF_17)]. | Clinical sepsis study patients [[17](file:///C:\Users\jld88\AppData\Local\Microsoft\Windows\Temporary%20Internet%20Files\Content.Outlook\6WEZMPQW\Appendix_on_model.docx#_ENREF_17)]. | - |
| $u_{min}$ | 16.7 mU/min | Minimum insulin secretion | Y | Constraint derived from lower range of clinical insulin secretion data [[17](file:///C:\Users\jld88\AppData\Local\Microsoft\Windows\Temporary%20Internet%20Files\Content.Outlook\6WEZMPQW\Appendix_on_model.docx#_ENREF_17)]. | Clinical sepsis study patients [[17](file:///C:\Users\jld88\AppData\Local\Microsoft\Windows\Temporary%20Internet%20Files\Content.Outlook\6WEZMPQW\Appendix_on_model.docx#_ENREF_17)]. | - |
| $u_{max}$ | 266.7 mU/min | Maximum insulin secretion | Y | Constraint derived from upper range of clinical insulin secretion data [[17](file:///C:\Users\jld88\AppData\Local\Microsoft\Windows\Temporary%20Internet%20Files\Content.Outlook\6WEZMPQW\Appendix_on_model.docx#_ENREF_17)]. | Clinical sepsis study patients [[17](file:///C:\Users\jld88\AppData\Local\Microsoft\Windows\Temporary%20Internet%20Files\Content.Outlook\6WEZMPQW\Appendix_on_model.docx#_ENREF_17)]. | - |
| $V_{I}$ | 4.0 L | Insulin distribution volume | Y | Chosen from literature [[17](file:///C:\Users\jld88\AppData\Local\Microsoft\Windows\Temporary%20Internet%20Files\Content.Outlook\6WEZMPQW\Appendix_on_model.docx#_ENREF_17)]. | Literature: [[9](file:///C:\Users\jld88\AppData\Local\Microsoft\Windows\Temporary%20Internet%20Files\Content.Outlook\6WEZMPQW\Appendix_on_model.docx#_ENREF_9), [62](file:///C:\Users\jld88\AppData\Local\Microsoft\Windows\Temporary%20Internet%20Files\Content.Outlook\6WEZMPQW\Appendix_on_model.docx#_ENREF_62)] | 3.15 – 4.75 L |

**Table A1-2**. Exogenous input variables to the glucose-insulin model.

| **Variable** | **Unit** | **Description** |
| --- | --- | --- |
| PN(t) | mmol/min | Intravenous glucose input rate (parenteral nutrition) |
| D(t) | mmol/min | Oral glucose input rate (enteral nutrition) |
| u_ex_(t) | mU/min | intravenous insulin input rate |

The insulin sensitivity SI can be identified hourly from blood glucose data along with the clinical insulin and nutritional inputs from all sources [[11](#_ENREF_11), [12](#_ENREF_12)]. SI is also the critical parameter in predicting the outcome of a nutrition and/or insulin intervention in this model, based on the definition above [[2](#_ENREF_2), [3](#_ENREF_3), [11](#_ENREF_11)]. It represents the whole body balance of insulin and carbohydrate from all sources. SI can vary with patient-status hour to hour, with larger acute changes or smaller gradual evolution. Thus, the identified SI can be used to characterise metabolic response and evolution for cohorts or specific-patients, enabling more optimal and robust dosing [[19-22](#_ENREF_19)]. Two example SI profiles and model fit to clinical data can be found in Figure A1-2. Both show stable BG within the 4.4 – 8.0 mmol/L range, despite different underlying insulin sensitivity variability and the insulin and nutrition doses required to achieve comparable BG stability.

*
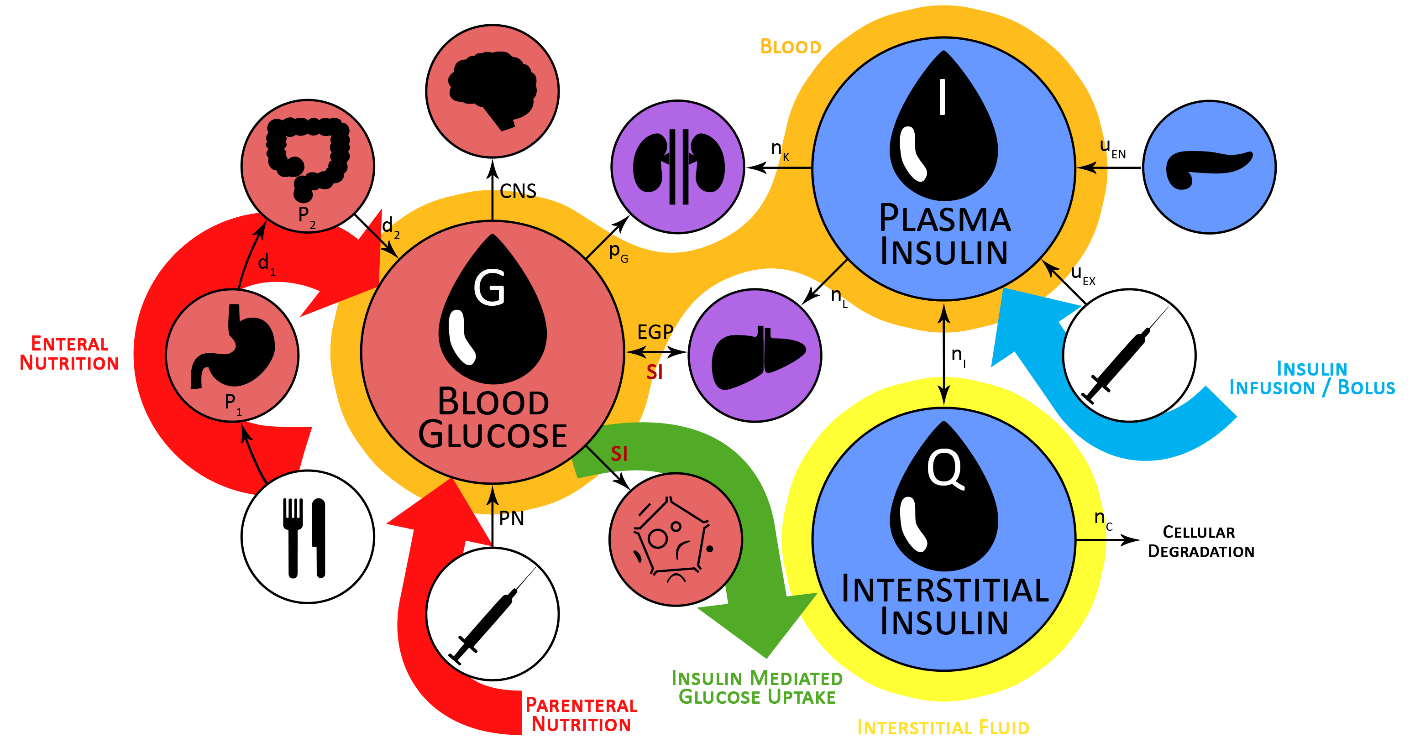
***Figure A1-1**: Model schematic for Equations (1)-(3) showing the physiological compartments and clearances, as well as the appearance of exogenous insulin and carbohydrate, and their kinetic pathways. Insulin sensitivity (SI) can vary over time (hour to hour) thus affecting glycaemic outcomes for a given insulin and/or nutrition intervention.

**Figure A1-2**: Example patients from clinical data, showing measured blood glucose (BG), clinically delivered insulin and nutrition, and model fitted insulin sensitivity (SI). A more ‘Stable’ SI profile (left) and more variable SI profile (right).

**Additional File 1 References**:

1. Chase JG, Shaw GM, Lotz T, LeCompte A, Wong J, Lin J, Lonergan T, Willacy M, Hann CE: **Model-based insulin and nutrition administration for tight glycaemic control in critical care**. *Curr Drug Deliv* 2007, **4**(4):283-296.

2. Chase JG, Shaw GM, Lin J, Doran CV, Hann C, Robertson MB, Browne PM, Lotz T, Wake GC, Broughton B: **Adaptive bolus-based targeted glucose regulation of hyperglycaemia in critical care**. *Med Eng Phys* 2005, **27**(1):1-11.

3. Chase JG, Shaw GM, Lin J, Doran CV, Hann C, Lotz T, Wake GC, Broughton B: **Targeted glycemic reduction in critical care using closed-loop control**. *Diabetes Technol Ther* 2005, **7**(2):274-282.

4. Wong XW, Singh-Levett I, Hollingsworth LJ, Shaw GM, Hann CE, Lotz T, Lin J, Wong OS, Chase JG: **A novel, model-based insulin and nutrition delivery controller for glycemic regulation in critically ill patients**. *Diabetes Technol Ther* 2006, **8**(2):174-190.

5. Wong XW, Chase JG, Shaw GM, Hann CE, Lotz T, Lin J, Singh-Levett I, Hollingsworth LJ, Wong OS, Andreassen S: **Model predictive glycaemic regulation in critical illness using insulin and nutrition input: a pilot study**. *Med Eng Phys* 2006, **28**(7):665-681.

6. Chase JG, Wong X-W, Singh-Levett I, Hollingsworth LJ, Hann CE, Shaw GM, Lotz T, Lin J: **Simulation and initial proof-of-concept validation of a glycaemic regulation algorithm in critical care**. *Control Engineering Practice* 2008, **16**(3):271-285

7. Lonergan T, LeCompte A, Willacy M, Chase JG, Shaw GM, Wong XW, Lotz T, Lin J, Hann CE: **A simple insulin-nutrition protocol for tight glycemic control in critical illness: development and protocol comparison**. *Diabetes Technol Ther* 2006, **8**(2):191-206.

8. Lonergan T, Le Compte A, Willacy M, Chase JG, Shaw GM, Hann CE, Lotz T, Lin J, Wong XW: **A pilot study of the SPRINT protocol for tight glycemic control in critically Ill patients**. *Diabetes Technol Ther* 2006, **8**(4):449-462.

9. Suhaimi F, Le Compte A, Preiser JC, Shaw GM, Massion P, Radermecker R, Pretty C, Lin J, Desaive T, Chase JG: **What Makes Tight Glycemic Control (TGC) Tight? The impact of variability and nutrition in 2 clinical studies**. *Journal of Diabetes Science and Technology* 2010, **4**(2):284-298.

10. Chase JG, Suhaimi F, Penning S, Preiser JC, Le Compte AJ, Lin J, Pretty CG, Shaw GM, Moorhead KT, Desaive T: **Validation of a model-based virtual trials method for tight glycemic control in intensive care**. *Biomed Eng Online* 2010, **9**:84.

11. Hann CE, Chase JG, Lin J, Lotz T, Doran CV, Shaw GM: **Integral-based parameter identification for long-term dynamic verification of a glucose-insulin system model**. *Comput Methods Programs Biomed* 2005, **77**(3):259-270.

12. Hann C, Chase J, Ypma M, Elfring J, Nor N, Lawrence P, Shaw G: **The Impact of Parameter Identification Methods on Drug Therapy Control in an Intensive Care Unit**. *The Open Medical Informatics Journal* 2008, **2**:92-104.

13. Cobelli C, Carson ER, Finkelstein L, Leaning MS: **Validation of simple and complex models in physiology and medicine**. *Am J Physiol* 1984, **246**(2 Pt 2):R259-266.

14. Cobelli C, Pacini G, Toffolo G, Sacca L: **Estimation of insulin sensitivity and glucose clearance from minimal model: new insights from labeled IVGTT**. *Am J Physiol* 1986, **250**(5 Pt 1):E591-598.

15. Carson ER, Cobelli C: **Modelling methodology for physiology and medicine**. San Diego: Academic Press; 2001.

16. Cobelli C, Caumo A, Omenetto M: **Minimal model SG overestimation and SI underestimation: improved accuracy by a Bayesian two-compartment model**. *Am J Physiol* 1999, **277**(3 Pt 1):E481-488.

17. Hovorka R, Chassin LJ, Ellmerer M, Plank J, Wilinska ME: **A simulation model of glucose regulation in the critically ill**. *Physiol Meas* 2008, **29**(8):959-978.

18. Pillonetto G, Sparacino G, Cobelli C: **Numerical non-identifiability regions of the minimal model of glucose kinetics: superiority of Bayesian estimation**. *Math Biosci* 2003, **184**(1):53-67.

19. Le Compte A, Chase J, Lynn A, Hann C, Shaw G, Wong X, Lin J: **Blood Glucose Controller for Neonatal Intensive Care: Virtual trials development and 1st clinical trials**. *Journal of Diabetes Science and Technology (JoDST)* 2009, **3**(5):1066-1081.

20. Le Compte AJ, Lee DS, Chase JG, Lin J, Lynn A, Shaw GM: **Blood glucose prediction using stochastic modeling in neonatal intensive care**. *IEEE Trans Biomed Eng* 2010, **57**(3):509-518.

21. Lin J, Lee D, Chase J, Hann C, Lotz T, Wong X: **Stochastic Modelling of Insulin Sensitivity Variability in Critical Care**. *Biomedical Signal Processing & Control* 2006, **1**:229-242.

22. Lin J, Lee D, Chase JG, Shaw GM, Le Compte A, Lotz T, Wong J, Lonergan T, Hann CE: **Stochastic modelling of insulin sensitivity and adaptive glycemic control for critical care**. *Comput Methods Programs Biomed* 2008, **89**(2):141-152.

23. Lotz TF, Chase JG, McAuley KA, Lee DS, Lin J, Hann CE, Mann JI: **Transient and steady-state euglycemic clamp validation of a model for glycemic control and insulin sensitivity testing**. *Diabetes Technol Ther* 2006, **8**(3):338-346.

24. Lotz T: **High Resolution Clinical Model-Based Assessment of Insulin Sensitivity**. Christchurch: University of Canterbury; 2007.

25. Lotz TF, Chase JG, McAuley KA, Shaw GM, Wong XW, Lin J, Lecompte A, Hann CE, Mann JI: **Monte Carlo analysis of a new model-based method for insulin sensitivity testing**. *Comput Methods Programs Biomed* 2008, **89**(3):215-225.

26. Lin J, Razak NN, Pretty CG, Le Compte A, Docherty P, Parente JD, Shaw GM, Hann CE, Geoffrey Chase J: **A physiological Intensive Control Insulin-Nutrition-Glucose (ICING) model validated in critically ill patients**. *Comput Methods Programs Biomed* 2011, **Epub ahead of print**.

27. Lin J, Lee D, Chase JG, Shaw GM, Le Compte A, Lotz T, Wong J, Lonergan T, Hann CE: **Stochastic modelling of insulin sensitivity and adaptive glycemic control for critical care**. *Computer Methods and Programs in Biomedicine* 2008, **89**(2):141-152.

28. Preiser JC, Devos P, Ruiz-Santana S, Melot C, Annane D, Groeneveld J, Iapichino G, Leverve X, Nitenberg G, Singer P *et al*: **A prospective randomised multi-centre controlled trial on tight glucose control by intensive insulin therapy in adult intensive care units: the Glucontrol study**. *Intensive Care Med* 2009, **35**(10):1738-1748.

29. McAuley KA, Williams SM, Mann JI, Goulding A, Chisholm A, Wilson N, Story G, McLay RT, Harper MJ, Jones IE: **Intensive lifestyle changes are necessary to improve insulin sensitivity: a randomized controlled trial**. *Diabetes Care* 2002, **25**(3):445-452.

30. Chase JG, Shaw G, Le Compte A, Lonergan T, Willacy M, Wong X-W, Lin J, Lotz T, Lee D, Hann C: **Implementation and evaluation of the SPRINT protocol for tight glycaemic control in critically ill patients: a clinical practice change**. *Critical Care* 2008, **12**(2):R49.

31. Le Compte A: **Modelling the Glucose-Insulin Regulatory System for Glycaemic Control in Neonatal Intensive Care**. *PhD thesis.* Christchurch, New Zealand: University of Canterbury; 2009.

32. Lin J: **Robust Modelling and Control of the Glucose-Insulin Regulatory System for Tight Glycemic Control of Critical Care Patients**. Christchurch: University of Canterbury; 2007.
