# Supplementary material for: Untangling glycaemia and mortality in critical care
Source: Crit Care. 2017 Jun 24;21:152. doi: 10.1186/s13054-017-1725-y (PMC5482947; doi:10.1186/s13054-017-1725-y)
Supplement: Supplementary file 2 — Clinical significance calculations. This file presents the underlying calculations used to define the clinical ‘equivalence range’ used in this study. (DOCX 68 kb) [file 13054_2017_1725_MOESM2_ESM.docx]

**Additional File 2: Clinical Significance Calculations**

The equivalence range is determined by examining the changes in insulin sensitivity (SI) which become clinically significant. This is different from hypothesis testing, as the equivalence range is determined independently. Further, in theory, two samples can be clinically equivalent, even if they are statistically different. The equivalence range for insulin sensitivity changes can be determined in two ways:

- Change in SI possible due to measurement error
- Change in SI required to change the insulin dose recommendation

The first accounts for variation in model-based SI during the identification process due to measurement error, while the second examines its impact on glycaemic control outputs. The equivalence range will be determined conservatively as the minimum changes in SI required to cause clinically significant change, to ensure the strongest test of equivalence.

A2.1 Change in SI due to measurement error:

Model-based blood glucose is defined:

| $\dot{G}(t)=-p_{G}G(t)- S_{I}\left( t \right)G(t)\frac{Q(t)}{1+\alpha_{G}Q(t)}+\frac{P\left( t \right)+EGP-CNS}{V_{G}}$ | (1) |
| --- | --- |

Parameter descriptions and values can be found in Table A2.1. Considering the average response over an hour, Equation 1 can be rewritten:

| $\frac{\Delta G}{60min}=-p_{G}G_{m}- S_{I}G_{m}\frac{Q_{m}}{1+\alpha_{G}Q_{m}}+\frac{P+EGP-CNS}{V_{G}}$ | (2) |
| --- | --- |

If Equation 2 is then re-arranged for SI:

| $S_{I}= \frac{\left( -p_{G}G_{m}+\left( P+EGP-CNS \right)/{V_{G}}- {\Delta G}/{60min} \right)\left( 1+\alpha_{G}Q_{m} \right)}{G_{m}Q_{m}}$ | (3) |
| --- | --- |

For an absolute error in BG of ξ %, according to Equation 3 the new SI becomes

| $S_{I,\xi}= \frac{\left( -p_{G}G_{m}(1+\xi/{100})+\left( P+EGP-CNS \right)/{V_{G}}- {\Delta G}/{60min} \right)\left( 1+\alpha_{G}Q_{m} \right)}{Q_{m}G_{m}(1+\xi/{100})}$ | (4) |
| --- | --- |

The possible percentage difference in SI due to measurement error is then:

| ${\Delta S}_{I,\xi}=\frac{S_{I,\xi}- S_{I}}{S_{I}}$ | (5) |
| --- | --- |

For the Arkray Glucocard X, a very similar device compared to the Super Glucocard II, the standard deviation of percentage error on a BG measurement is 9.4% [1]. From Equation 4, the insulin sensitivity can be affected by insulin and nutrition inputs (indirectly reflected through $Q_{m}$ and $P$ respectively), as well as the range of change of BG. The range of possible ${\Delta S}_{I,\xi}$ for different $G_{m}$ values, $Q_{m}$ values, ${\Delta G}/{60min}$, and nutrition inputs ($P$) was assessed.

**Table A2.1 Glucose Parameter Values and Description**

| **Parameter** | **Value and/or Units** | **Description** |
| --- | --- | --- |
| *G(t)* | mmol/l | Blood glucose concentration |
| *Q(t)* | mU/l | Interstitial insulin concentration |
| *S_I_(t)* | l/mU/min | Insulin sensitivity |
| $-p_{G}$ | 0.006/min | Kidney and general non-insulin mediated clearance |
| $\alpha_{G}$ | 1/65 l/mU | Saturation of insulin-mediated glucose uptake |
| $V_{G}$ | 13.3 L | Glucose distribution volume |
| $EGP$ | 1.16 mmol/min | Endogenous glucose production (hepatic) |
| $CNS$ | 0.3 mmol/min | Glucose uptake by central nervous system |

***Nutrition range (P)***:

Assuming a goal feed of 2000 kCal/day (25 kCal/kg/day for an 80kg individual) of Glucerna (1.0 kCal/mL, 0.0812 g/mL glucose), which is the primary feed type in this unit and study, goal feed (rounded to the nearest 5mL/hr) is 85 mL/hr (38.3 mmol/hr glucose). The range of nutrition tested was 50:10:120% of this feed target.

Sensitivity to nutrition inputs is shown in Figure A2.1. As the nutrition increases, the percent change in SI required to account for glucometer error decreases due to higher overall insulin sensitivity.

**Figure A2.1:** Effect of nutrition inputs on insulin sensitivity (SI) and the potential difference in SI due to BG measurement error. Nutrition was modulated between 50 and 120% of the clinical goal (2000 kCal/day).

***Plasma insulin range (****Q_m_****)***:

Sensitivity to *Q_m_* within the common range (10 – 100 mU/L) was tested, and results are shown in Figure A2.2. The percentage difference in SI is not sensitive to *Q_m_*, as it normalises in Equation 5. The case where a change in *Q_m_* is causing the change in SI, rather than glucometer error as in Equations 4 and 5, is examined later in section A2.3.

**Figure A2.2:** Effect of nutrition inputs on insulin sensitivity (SI) and the potential difference in SI due to BG measurement error. Interstitial insulin (Q) was modulated between 5 – 50 mU/L, which is a commonly observed range.

***Rate of Change of Blood Glucose (ΔBG/60min)***

Sensitivity to hourly changes in BG were examined, with this change ranging from an absolute change in BG of 2 mmol/L (a relatively large hourly change) to no change in BG. Results in Figure A2.3 show that the minimal change in SI required to account for glucometer error occurs at steady state (no change in BG). It is thus the most conservative case.

**Figure A2.3:** Effect of nutrition inputs on insulin sensitivity (SI) and the potential difference in SI due to BG measurement error. The change in BG over an hour was modulated between an absolute change of 2 mmol/L (a relatively large change) and no change.

The percentage change in SI required to account for glucometer error (ξ in %) is shown for a range of different error magnitudes in Figure A2.4. As the percentage or CV of glucometer error increases, the larger the clinically significant change in SI.

**Figure A2.4:** Effect of the magnitude of measurement error on the potential difference in SI due to BG measurement error.

A2.2 Change in SI required to change an insulin intervention:

The plasma and interstitial insulin model equations are defined:

| $\dot{I}\left( t \right)=-n_{K}I(t)-n_{L}\frac{I(t)}{1+\alpha_{I}I(t)}-n_{I}\left( I(t)-Q(t) \right)+\frac{u_{ex}\left( t \right)}{V_{I}}+\left( 1-x_{L} \right)\frac{u_{en}(G)}{V_{I}}$ | (6) |
| --- | --- |
| $\dot{Q}(t)=n_{I}(I(t)-Q(t))-n_{C}\frac{Q(t)}{1+\alpha_{G}Q(t)}$ | (7) |

Parameter descriptions and values are given in Table A2.2.

**Table A2.2 Insulin Model Parameter Values and Description**

| **Parameter** | **Value and/or Units** | **Description** |
| --- | --- | --- |
| *I(t)* | mU/l | Plasma insulin concentration |
| *Q(t)* | mU/l | Interstitial insulin concentration |
| $x_{L}$ | 0.67 | Fractional first pass hepatics insulin clearance from portal vein |
| $n_{L}$ | 0.1578 min^-1^ | Rate parameter: general hepatic insulin clearance |
| $\alpha_{I}$ | 1.7x10-3 l/mU | Saturation of hepatics insulin clearance |
| $n_{K}$ | 0.0542 min^-1^ | Rate parameter: kidney clearance of insulin |
| $n_{C}$ | 0.006 min^-1^ | Rate parameter: cellular degradation of internalised insulin |
| $n_{I}$ | 0.006 min^-1^ | Rate parameter: diffusion of insulin between plasma and interstitium |
| $u_{en}$ | mU/min | Pancreatic insulin secretion |
| $V_{I}$ | 4.0 L | Insulin distribution volume |

Assuming steady state:

| $0=-n_{K}I_{ss}-n_{L}\frac{I_{ss}}{1+\alpha_{I}I_{ss}}-n_{I}\left( I_{ss}-Q_{ss} \right)+\frac{u_{ex}\left( t \right)}{V_{I}}+\left( 1-x_{L} \right)\frac{u_{en}(G)}{V_{I}}$ | (8) |
| --- | --- |
| $0=n_{I}(I_{ss}-Q_{ss})-n_{C}\frac{Q_{ss}}{1+\alpha_{G}Q_{ss}}$ | (9) |

Equations 8 and 9 can be numerically solved (Newton’s method was used here) for Qss and Iss for a given insulin dose, $u_{ex}$. As insulin secretion, $u_{en}$, is dependent on BG, this steady state will depend on the current BG level.

The change in insulin sensitivity required to change an insulin dose by 1 U, given a constant BG, can be estimated from the insulin-mediated glucose uptake component of Equation 1. The minimal degree of allowable difference in SI for a clinical change in insulin treatments can be estimated from insulin-mediated glucose uptake and the assumption of no change in BG:

| $S_{I,1}G\frac{Q_{SS,1}}{1+\alpha_{G}Q_{SS,1}}= S_{I,2}G\frac{Q_{SS,2}}{1+\alpha_{G}Q_{SS,2}}$ | (10) |
| --- | --- |
| $\frac{S_{I,2}}{S_{I,1}}= \frac{Q_{SS,1}}{Q_{SS,2}}\frac{\left( 1+\alpha_{G}Q_{SS,2} \right)}{\left( 1+\alpha_{G}Q_{SS,1} \right)}$ | (11) |

The estimation of the minimal percentage change in SI required for a change in insulin intervention is shown in Figure A2.5. At higher insulin doses, Qss is higher and a 1 U change in insulin dose represents a smaller fraction of Qss. As a result, the percentage change in SI that allows a change in intervention narrows. Thus, the control system is more robust to error or variability in SI at lower insulin doses, which are more typical once BG is lowered into the normal range.

**Figure A2.5: Model-based estimates of steady state interstitial insulin concentration for a given insulin dose, and the minimum percentage change in SI required to change an insulin intervention by 1U, assuming steady state Insulin.**

*A2.3 Summary and recommendation for equivalence:*

For a typical error standard deviation on Glucocard glucometers of 9.4%, to be conservative within the range of commonly observed BG (4.0 – 10.0 mmol/L) the minimum difference in SI that would be clinically significant beyond glucometer error is ~ 12-15% (Figures A2.1 – A2.3), and a function of BG. In the case of sustained hyperglycaemia, SI tends to be low, so percentage changes in SI required for clinical significance are higher.

Hence, equations 4 and 5 will be used to determine the equivalence range, using 100% goal feed and a conservative estimate of no change in blood glucose. The resulting plot is in Figure A2.6. For equivalence the 90% confidence interval (CI) must lie within the defined equivalence interval.

**Figure A2.6: Equivalence range for insulin sensitivity (SI), using a conservative estimation of no change in blood glucose level (steady state). For non-steady state conditions, this range widens.**

**Additional File 2 References:**

1. Freckmann G, Baumstark A, Jendrike N, Zschornack E, Kocher S, Tshiananga J, Heister F, Haug C: **System accuracy evaluation of 27 blood glucose monitoring systems according to DIN EN ISO 15197.** *Diabetes Technol Ther* 2010, **12:**221-231.
